# Coupling Cellular Automata and a Genetic Algorithm to Generate a Vibrant Urban Form—A Case Study of Wuhan, China

**DOI:** 10.3390/ijerph182111013

**Published:** 2021-10-20

**Authors:** Renyang Wang, Qingsong He, Lu Zhang, Huiying Wang

**Affiliations:** 1 Research Institute of New Economic, Ningbo University of Finance & Economics, Ningbo 315175, China; wangrenyang@nbufe.edu.cn; 2 College of Public Administration, Huazhong University of Science and Technology, Wuhan 430074, China; heqingsong@hust.edu.cn; 3 School of Public Administration, Central China Normal University, Wuhan 430079, China; 4 School of Government, University of International Business and Economics, Beijing 100029, China

**Keywords:** urban vitality, urban form, urban growth pattern, cellular automata, genetic algorithm, Wuhan

## Abstract

Enhancing urban vitality is a key goal for both the government and ordinary urban residents, and creating this vitality is emphasized in China’s urban development strategy. Enhancing urban vitality through the rational design of urban forms is a leading topic of Western urban research. An urban growth pattern (UGP) reflects the dual characteristics of a static pattern and the dynamic evolution of the external urban form. It affects urban vitality by influencing the spatial allocation of internal structural elements and patterns in the adjacent location. The cellular automata (CA) mode can effectively simulate the aggregation process of urban growth (infilling expansion or edge expansion). However, it does not simulate the diffusion of urban growth, specifically the evolution of outlying expansion. In addition, CA focuses on learning, simulating, and building knowledge about geographic processes, but does not spatially optimize collaborative land use against multiple objectives or model multi-scale land use. As such, this paper applies a coupling model called the “promoting urban vitality model,” based on cellular automata (CA) and genetic algorithm (GA) (abbreviated as UV-CAGA). UV-CAGA optimally allocates cells with different UGPs, creating a city form that promotes urban vitality. Wuhan, the largest city in Central China, was selected as a case study to simulate and optimize its urban morphology for 2025. The main findings were as follows. (1) The urban vitality of the optimized urban form scheme was 4.8% higher than the simulated natural expansion scheme. (2) Compared to 2015, after optimization, the simulated sizes of the newly increased outlying, edge, and infilling areas in 2025 were 6.51 km^2^, 102.69 km^2^, and 23.48 km^2^, respectively; these increases accounted for 4.90%, 77.32%, and 17.68%, respectively, of the newly increased construction land area. This indicated that Wuhan is expected to have a very compact urban form. (3) The infilling expansion type resulted in the highest average urban vitality level (0.215); the edge expansion type had the second highest level (0.206); outlying growth achieved the lowest vitality level (0.199). The UV-CAGA model proposed in this paper improves on existing geographical process simulation and spatial optimization models. The study successfully couples the “bottom-up” CA model and “top-down” genetic algorithm to generate dynamic urban form optimization simulations. This significantly improves upon traditional CA models, which do not simulate the “diffusion” process. At the same time, the spatial optimization framework of the genetic algorithm in the model also provides insights related to other effects related to urban form optimization, such as urban environmental security, commuting, and air pollution. The integration of related research is expected to enrich and improve urban planning tools and improve the topic’s scientific foundation.

## 1. Introduction

Urban vitality is the foundation for urban attractiveness and the most direct external expression of comprehensive urban strength. Urban vitality is an abstract concept, with no unified definition [1]. J. Jacobs first proposed the concept, noting that complex human activities and human life create the diversity of urban life, and that the expression of urban life is urban vitality [2]. In the book “Good urban form,” Lynch positioned “vitality” as the primary standard to evaluate the quality of an urban form, defining it as the degree of settlement support for life functions, ecological requirements, and human activities [3]. Urban vitality is understood to be the diversity of urban life generated by people’s gatherings and activities. It is widely considered to be a spatial feature and its isomorphism with human activities is its main social attribute [4,5].

Urban form refers to a spatial system composed of structure (spatial arrangement of elements), shape (spatial outline outside the city), and their mutual relationships [6]. As the external representation of urban spatial structure, the urban form is a spatial result of the comprehensive action of different natural, social, and economic factors on the city [7]. Whether the form is reasonable or not will impact production and living, the environment, and the traffic of the city [8]. The correlation between urban form and commuting efficiency [9,10], surface temperature [11,12,13], air quality [14,15,16], and local urban and climate environment [17,18,19,20] has been widely studied. The international debate on urban form has gained an even higher importance since the 2020 pandemic outbreak, particularly with respect to emphasizing the right to city access, the development of the green city, and the proximity of resources that will change the urban form [21,22,23,24]. The influence of urban form on urban vitality comes from the location, function, transportation, form, and landscape [25,26]. Promoting urban vitality through urban form design has become an important issue since the birth of New Urbanism in the West [27,28], and scholars have conducted significant preliminary research in this field [1,2,3,4,5,29]. Traditional social survey data or statistical data based on administrative boundaries focus on the internal spatial design at the street or block scale, but do not consider the optimization of external macro urban form patterns [1,25]. Many types of imagery data from different sensors have emerged in the era of information communication technology. The continuous emergence of multi-source and big geographic data facilitates the monitoring of the evolution of urban form at multiple scales. The fine-grained observation of urban vitality has increasingly attracted the attention of urban researchers [5,25,30].

Many studies have explored the relationship between urban form and urban vitality; however, most have not considered the temporal attribute of space [1,25]. Academics generally refer to the urban form as the static characteristics of urban structure, without reflecting the process of urban evolution. This process constantly changes the urban structure, and China’s cities will continue to evolve and expand in the future. However, it is difficult to grasp the impact of the evolution of urban form on urban vitality. We believe only one study [25] has considered the impact of such growth patterns on urban vitality. That study provided an “associated result,” and data mining was not further applied to develop an optimized simulation model that promoted vitality. In addition, that study did not assess the impacts of spatial proximity (location conditions) and other static distribution pattern indicators on urban vitality.

By 2030, China’s urbanization rate is expected to reach approximately 70%, and in the next ten years, approximately 160 million people are expected to move to cities. Continuous urban expansion, through the occupation of surrounding non-construction land, construction of outlying development parks, internal urban village reconstruction, and urban renewal will continuously change the urban form. This will occur through three types of evolution: infilling, outlying, and edge. Each type has different impacts on urban internal structure, land use, and traffic layout, leading to different effects on urban vitality. Infilling growth is intensive and compact, providing more urban functions on limited land. It promotes urban high-rise buildings, improves mixed land use, and promotes the enrichment of urban infrastructure. These changes in urban form and structure significantly affect urban vitality [7,31,32]. The second type of expansion, outlying development, significantly reduces land use costs [33]. New development zones or industrial parks in many cities currently belong to this type [1]. In general, these newly developed urban areas have a single land use type, high vacancy rates for residential land, poor infrastructure, low population density, and abandoned commercial activities [29]. However, in China, several new enclave areas have a higher development intensity and population density than the original urban areas, forming more vibrant areas [1] Third, the edge type is the main type of urban growth in China [7]. Data mining to assess the association between the UGP of construction land across China and urban vitality in one study [25] revealed that edge development in new urban areas had a strong relationship with a high population concentration, high housing prices, and mixed land functions.

Academics have debated about the types of urban forms that promote urban vitality. Influenced by different planning theories, Western scholars call for and praise the development of the “compact city” [2,34,35]. However, other scholars question or oppose the pursuit of the compact urban form, arguing that responsible diffusion can promote urban vitality [25,36]. This highlights the need to analyze and discuss the role of “compact” or “diffuse” urban forms in urban development. In the context of Chinese cities, it is important to balance the two forms in terms of quantity and spatial allocation [7]. Previous studies have not yet simulated the “diffusion” process in microcosmic space, nor have they determined the optimal combination of urban form evolution and spatial distribution at a macro scale, to promote urban vitality. As such, further modeling and theoretical work are needed.

Optimizing the urban form to promote urban vitality is a complex non-linear, unstructured, multi-agent, multi-objective, and multi-scale coordinated spatial optimization problem. It involves spatial optimization allocation, such as the unit, optimization objectives, optimization limiting factors, optimization operations, and optimization scheme optimization. Further, the problem is difficult to solve using traditional linear programming methods. In recent years, the heuristic algorithm represented by genetic algorithm (GA) has provided a “top-down” optimization computing framework, which helps realize different optimal objectives and which provides a computing technology for addressing nonlinear, high-dimensional, unstructured, and other complex problems. GA has been applied to land use spatial optimization problems [37,38]. However, GA is a pure mathematical method, lacking spatial knowledge and optimization constraints, and does not easily reflect decision making related to spatial change on a micro scale. The approach also does not effectively clarify the internal mechanism and rules of spatial evolution on the micro scale. As such, it cannot on its own achieve a reasonable spatial optimal allocation scheme [39,40]. Cellular automata (CA) is a common spatial simulation model in the field of urban growth and is widely used because it can simulate complex time-space dynamic processes with simple rules [41]. CA provides a “bottom-up” spatial modeling framework for solving complex geographic problems. However, in the traditional urban CA model, to determine whether a non-urban cell changes or not, it needs to consider the state of its neighborhood cell. Usually, a cell is transformed into an urban cell only if there are enough urban cells in its neighborhood. The traditional CA model, based on Moore or Von Neumann neighborhood rules, can effectively simulate the aggregation process of urban growth (infilling expansion or edge expansion), but it cannot simulate the diffusion of urban growth, that is, it does not simulate the evolution of outlying expansion [7,37,38]. In addition, CA focuses on geographic process learning and simulation but does not spatially optimize multiple collaborative land use objectives and multi-scale land use [42]. Therefore, combining GA and CA may simultaneously optimize the evaluation of the evolution of urban form and spatial distribution in magnitude and space [1].

In the future, China’s urbanization will continue to change the urban form. Therefore, promoting urban vitality by optimizing urban form will become an important urban planning feature. Given this background, this paper first explores the relationship between urban form evolution, distribution patterns, and urban vitality using multi-source data and applies that relationship to a proposed UV-CAGA urban form simulation model. This provides theoretical and tool-based support for exploring the impacts of dynamic urban changes on urban vitality in the context of Chinese cities, promoting future vibrant urban forms and guiding cities towards healthy and sustainable development. Wuhan, the largest city in Central China, served as a research case to test the usability of the proposed model.

## 2. Materials and Methods

### 2.1. Study Area and Data

**Figure 1:**
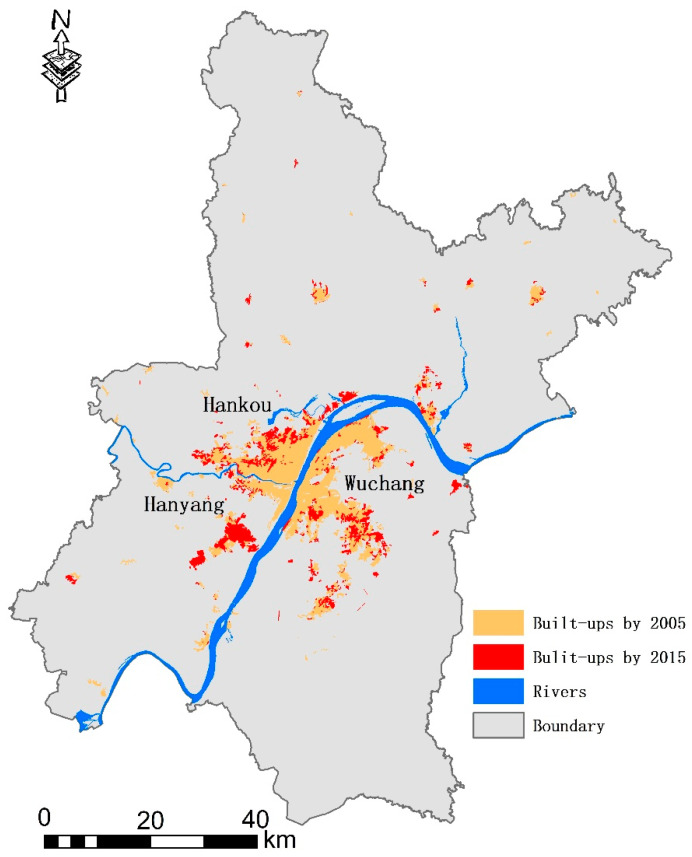
Study area.

Wuhan is located between 113°41′–115°05′ E longitude and 29°58′–31°22′ N latitude. The Yangtze River, the world’s third longest river, and its largest tributary, the Han River, run through the city center and divide the central city area into three parts. This forms a grid that includes Wuchang, Hankou, and Hanyang (Figure 1). By 2018, the city had 13 districts, covering a total area of 8494.41 km^2^, with a permanent resident population of 11.08 million and an urban population of 8.90 million. The urbanization rate was 80.20%, making it the largest city in central China. Wuhan is an important transportation hub and talent gathering place. The railway network connects both north and south and east and west. Wuhan is an important scientific research and education base, with many university resources. Its comprehensive strength in science and education ranks among the top in China.

We collected data from multiple sources about the study area, including land use data, basic geographic base map data, and open access data. The land use data came from the national land use/cover database of China (NLUD-C), produced by the Chinese Academy of Sciences. Its spatial resolution is 30 m using Landsat TM imagery and China–Brazil Earth Resources Satellite image data. Visual interpretation combined with field investigations ensured a classification accuracy of greater than 90% [7]. The construction land patches extracted from NLUD-C were used to identify urban areas. Urban vitality was evaluated using multi-source geographic data. Web crawler technology captured point of interest (POI) data, Weibo (the Chinese version of Facebook) check-in data, location-based public comment data, and residential house price data covering the research area. Landscan spatial population distribution data were collected at a 1 km resolution. OpenStreetMap road networks and night light remote sensing data were collected using a free download application. All geographic data were corrected (where needed) and validated before use. The base geographical conditions map was used to provide auxiliary data, including a vector map of administrative divisions in the research area, main rivers/lakes, main roads, the spatial locations of government residences, and other basic geographic information data.

### 2.2. UV-CAGA Steps

For this study, the GA numerical optimization capability was used to optimize the quantitative structure of cells representing different UGPs. The spatial layout was then determined using a CA spatial simulation. CA requires that the three types of urban spatial growth be simultaneously simulated to predict the spatial location and number of regions, given different future UGP evolutions. The approach uses probability modeling for different types of UGP evolution. GA includes the mapping between the elements and cellular space, crossover schemes, a mutation and selection operator, and determination of the objective function. Before detailing the methodology, this article defines urban vitality as the ability to form clusters of commercial activities and populations through spatial design. Diverse commercial activities and population agglomeration [4,43,44] are the most frequent features when describing urban vitality.

#### 2.2.1. CA to Determine Urban Growth Patterns for Newly Added Built-Up Cells

The UV-CAGA model identified the UGP of each newly added construction land cell during the operation. Because three types of growth were available, the simulation selected the most likely type based on its “probability.” We used logistic regression to determine the likelihood that each type would occur. Logistic regression is a probabilistic regression model, with class-based dependent variables. This approach is used to explore the relationship between a two- or multi-classification result Y (dependent variable) and the influencing factors X = (x1, x2, …, xn) (independent variable). In this application, Y was UGP, and the values were classified as 0 and 1. For any investigation, the value of 1 indicates a sample point belongs to that type; a value of 0 indicates it belongs to the other two types. For example, if the outlying type was being investigated, a certain number of points were randomly collected on the newly added construction land patches of that type, resulting in a Y value of 1. Points were also collected on the infilling and edge-type patches, resulting in a Y value of 0. The variable X is the driving factor influencing a different UGP.

Based on previous studies [38], we selected the following independent variables: distance to city center (Dis_center), distance to an existing built-up area (Dis_built), distance to a railway station (Dis_rail), distance to a water body (Dis_water), distance to a business center (Dis_business), population density (POPD), and road density (RD). These selections were based on previous studies [20]. The two new density indices do not directly affect UGP; however, they can indirectly affect UGP through the cost of land development [26]. For example, population density is related to the cost of demolition and resettlement. A higher population density means that the compensation input is higher. Areas with high road density tend to have sound infrastructure, reducing the extra cost of land development. All variables were tested for collinearity before use.

During the study’s sampling process, the UGP of newly added construction land patches was determined for a specific period: 2005–2015. UGP identification uses the widely applied Landscape Expansion Index (LEI), described in other studies [1,45]. Using this method, three logistic regression runs were conducted to generate the outlying, infilling, and edge probabilities for each cell and to determine the final type according to the roulette method [38]. Based on the UGP, we set different requirements for the number of urban cells in the CA neighborhood rule. For example, the number of urban cells in the original construction land in the outlying cell’s neighbor was 0. However, to keep the diffusional expansion from being too far from the city, we also restricted urban cells to be no more than 5 km distance from the original construction land. The edge type required two to four urban cells, while the infilling type required at least five urban cells of original construction land in the neighborhood.

#### 2.2.2. GA for Quantitative Optimization of Different Evolution Types

GA searches for optimal solutions by simulating natural evolutionary processes. To apply it to the space optimization problem, we first needed to establish a mapping relationship between GA elements and spatial elements. In this study, we mapped the cell space to the chromosome in the genetic algorithm; a single cell was expressed as the gene in the genetic algorithm. The three UGPs of the cell, defined as infilling, edge, and outlying, were encoded as 1, 2, and 3 in the gene, respectively. The genetic algorithm fitness function was defined as follows:

$$\mathrm{UV}=\mathrm{argmax}\overset{n}{\underset{i=1}{\sum }}\overset{k}{\underset{k=1}{\sum }}w_{ik}*x_{ik}$$

In this expression, UV represents comprehensive urban vitality; $x_{ik}$ represents the value of the k-th variable of the i-th newly added land cell; $w_{i}$ is its weight, which is determined by the coefficient of the regression model. The parameter N is the number of all new construction land cells. The goal of optimization is to continuously pursue a higher UV value.

The operational process of GA is as follows. The first step is to create the initial population, including multiple pairs of chromosomes (such as 100 pairs), set the number of crossover and mutation cells (such as 10), and determine the termination condition for iterations (reaching the planned area). Next, the mutation operator randomly selects several chromosomes and, then, randomly selects several non-construction land cells on the selected chromosomes and conducts type (coding) variation according to the probability of a cell’s evolution to different UGPs. Then, the crossover operator selects several cells on adjacent chromosomes for crossover using the roulette method. The next step is to calculate the fitness function value corresponding to the cell layout scheme on each chromosome and use the selection operator to preserve chromosomes with high fitness with a higher level of probability and enter the iteration of the next generation. These steps are then repeated. When the maximum fitness value for each generation is met and remains stable, the model operation is completed and the optimized scheme corresponding to the chromosome with the highest fitness value is generated.

#### 2.2.3. Factors Influencing Urban Vitality

In the UV-CAGA model, the evaluation function uses the sum of the urban vitality values for individual cells contained in the resulting urban form. The goal of the iterative process is a higher sum. This makes it necessary to estimate the vitality value of each newly added construction land cell. We used the multivariate regression model (MR) to implement this process. The dependent variable in MR is the viability value for each cell sample. The urban vitality assessment uses the indicators cited in other studies [1], including POI density (POID), land function mix (MIX), and sign-in density (CIQD) as proxies. POID reflects the abundance of urban infrastructure. Urban infrastructure is the infrastructure needed to ensure urban production, life activities, and urban survival and development. Infrastructure carries most kinds of traffic flow, material flow, population flow, and information flow generated by urban activities [1]. POI is an abstraction of infrastructure on the spatial map, and its density reflects the development of the overall infrastructure of the city [25]; the MIX affects different needs of urban residents, including work, entertainment, shopping, school, and other activities [25]. The rational mixed use of land supports intensive land use, industrial upgrades and transformations, and improvements in the efficiency of infrastructure utilization. Urban scholars often consider improvements in the mix of land functions as an important measure to promote urban vitality [2,46]. CIQD reflects the extent to which a local space attracts the attention of the population. It directly reflects the interaction between humans and urban space and provides a reference for monitoring the degree of urban commercial activity and its spatial distribution and development trends. It is a new measure of urban vitality [1]. A straightforward method, POID × MIX × CIQD, is used to evaluate urban vitality, while avoiding arbitrary or subjective settings for the weight of each dimension [29]. The main factors affecting vitality in this study were spatial proximity, socio-economic development, transportation connectivity, and UGP. Spatial proximity included location factors, such as the distance to the city center (${\mathrm{Dis}}_{\mathrm{center}}$) and distance to business centers (Wuhan’s five major business centers, ${\mathrm{Dis}}_{\mathrm{bcenter}}$). Socioeconomic factors included population density (PD) and GDP density (GDPD). Transportation connectivity included distance from public transportation stations (bus stops and subway stations, ${\mathrm{Dis}}_{\mathrm{station}}$) and road density (RD) within the buffer zone of a half-hour walk (2.5 km). UGP was used as a dummy variable.

Finally, we constructed the following regression equation:

$$\mathrm{UV}=f\left({\mathrm{Dis}}_{\mathrm{center}},{\mathrm{Dis}}_{\mathrm{bcenter}},\mathrm{PD},\mathrm{GDPD},{\mathrm{Dis}}_{\mathrm{station}},\mathrm{RD},\mathrm{UGP}\right)$$

In the above equation, f represents a multiple linear regression equation.

#### 2.2.4. Model Termination

The model was considered complete when the urban built-up area reached the simulated planning value. According to the Wuhan City 2035 plan, the area of the city is expected to continue to grow until 2025 and stabilize thereafter. This makes 2025 a deadline, based on the area listed for several years in Table 1. We established a linear relationship model for the year and area, which was Area = 24,331.2 + 12.3334 × Year (R2 = 0.92). It was forecasted that in 2025, there would be approximately 643.94 km^2^ of built-up areas.

## 3. Results and Analysis

### 3.1. Regression Analysis Results

**Figure 2:**
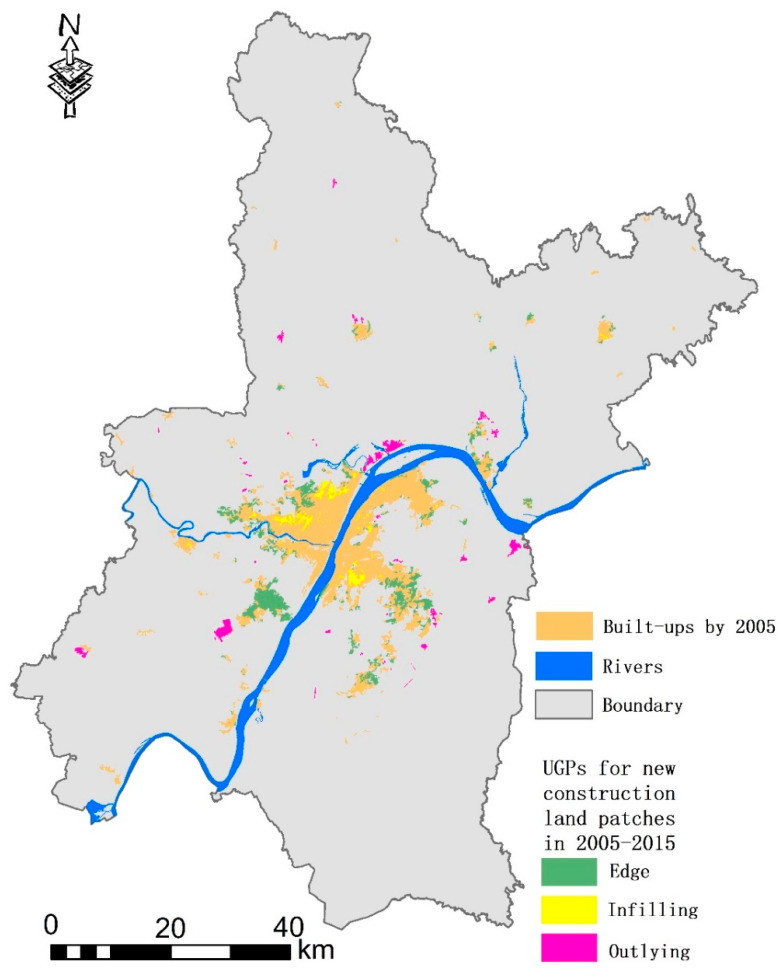
UGPs for new construction land patches in the study area from 2005 to 2015.

First, we identified the UGPs for new construction land patches in the study area from 2005 to 2015, as shown in Figure 2. The results showed that the outlying, edge, and infilling patch areas were 28.83 km^2^ (19.01%), 100.21 km^2^ (66.09%), and 22.59 km^2^ (14.90%), respectively. The results indicated that the edge type growth was the most important urban growth pattern in Wuhan during this period, followed by the outlying type that represented diffusion growth, followed by compact and intensive infilling growth.

We randomly selected 100 sample points from each type of patch. We used GIS to calculate the variable values at each sample point and, then, obtained the model variable parameters shown in Table 2. No variable had exactly the same positive or negative effects on the formation of the three types. For example, areas farther away from the city center and commercial centers were less favorable for infilling, but had a positive effect on edge and outlying growth. The distance from the existing built-up area had a strong positive relationship on outlying growth (4.569 ***), but not edge and infilling. The different effects of these three variables can be explained by the relationship between the spatial locations of the expansion types and the existing built-up areas. The findings that population agglomeration was associated with a higher probability of infilling or edge growth and that there was a lower correlation with outlying growth were similar to conclusions in a previous data mining study [8].

Distance from a railway only played a significant positive role in outlying growth, but not for edge and infilling. This may be because the role of a railway was as an external link rather than an internal link, and the enclave was an urban area outside the original built-up area. This type of area would be more likely to be affected by a railway. In contrast, infilling and edge types were extensions of the original construction land (internal and external), eliminating the need to consider railway impact. Road density had a significant negative effect on outlying growth, but a positive effect on edge and infilling. Roads connect the inner and outer parts of the city. A lower road density often indicates suboptimal city development, with a lot of land that has not been developed for construction land. This is similar to the gap between outlying regions and original construction land, i.e., undeveloped non-construction land.

However, outlying growth was isolated from the original construction land. Interconnections between the two were mainly through several main roads. Therefore, road density was significantly lower than the high density found inside the city and was significantly correlated with outlying growth. Both infilling and edge growth were associated with the original construction land space. In general, infilling patches were closer to the center of the city, where the road density was higher. This helps explain the positive relationship between high-density roads, infilling and edge growth, and why the coefficient for infilling growth exceeded that of edge growth (0.064 > 0.045). After obtaining the parameters above, we evaluated the probability that each newly added urban cell belonged to a different expansion type.

Based on the relationship between urban vitality and driving variables generated by the multiple regression analysis (Table 3), t ${\mathrm{Dis}}_{\mathrm{center}}$, ${\mathrm{Dis}}_{\mathrm{bcenter}}$, and GDPD had significant negative relationships with urban vitality: areas farther away from the urban and commercial centers with higher GDP had lower vitality levels. For every 1% unit increase in the value of ${\mathrm{Dis}}_{\mathrm{center}}$, ${\mathrm{Dis}}_{\mathrm{bcenter}}$, and GDP, urban vitality decreased by 0.114%, 0.198, and 0.044%, respectively. RD was significantly positively related to urban vitality: for every 1% unit increase in the value of RD, urban vitality increased by 3.859%. These results indicated that a superior location and good transportation facilities significantly promote urban vitality.

However, there was a surprising finding with respect to the relationship between GDPD and urban vitality. Our general expectation was that higher GDPD would lead to higher vitality. This is because of the focus of vitality on the ability to provide places to eat, drink, and play, as well as attractiveness (from a recreational perspective). However, the GDPD was lower compared to industrial parks and areas with several companies. Due to the few human activities and urban functions in these areas, there were few recreational facilities, so vitality was not as high.

In terms of UGP, different types of regression coefficients showed that outlying and edge types were not as effective in promoting urban vitality as infilling. This can also be considered from the location factor. In general, the distance between outlying, edge, and infilling areas decreased compared to urban and business centers, so the infilling area more easily radiated from urban and business centers. However, the convenient placement of living and working facilities near urban and business centers lead more people to gather and promote vitality.

### 3.2. Results of the Optimization Simulation

After continuously adjusting the UV-CAGA parameters, the population size in the model was finally set to 100; the numbers of mutations and intersections were 100; the termination condition for iteration was when the urban built-up area reached 643.9 km^2^ (equivalent to adding 51,994 built-up cells to the cell count in 2015). After about 650 iterations, the value of the evaluation function (total vitality) stabilized and no longer increased. We output the optimal configuration scheme with the highest vitality during the iteration process, along with the corresponding UGP distribution. The results showed that the average vitality of each cell after optimization was 0.201 standard units; this was approximately 94.50% of the average vitality of each cell for the original construction land. This was because the new urban area had a less complete infrastructure and service capabilities for employment and living than the original area and did not have as many advantages as the original city for attracting the population [8,25]. Statistics for different UGP compositions on the newly increased 132.8 km^2^ construction land showed that the outlying, edge, and infilling areas experienced increases of 6.51 km^2^, 102.69 km^2^, and 23.48 km^2^, respectively, accounting for 4.90%, 77.32%, and 17.68% of the growth, respectively.

These results highlighted two important dimensions. First, the change in the urban form of Wuhan completed urban expansion in a way that was consistent with the approach described in urban growth phase theory, which argues that urban growth moves from diffusion to convergence. The urban form of Wuhan converged [25,38]. The diffusion process represented by outlying growth made up a small proportion of the total growth. Second, outlying growth did not significantly promote urban vitality in the same way that edge and infilling growth did. This aligns with the academic community’s call to promote urban vitality with compact urban development [2,25,26]. This inference is supported by the fact that during the GA process of “searching for optimization” (i.e., promoting higher vitality), the outlying type was continuously eliminated, as it could not improve overall vitality. This resulted in that type of expansion contributing only a very low proportion of the growth.

**Figure 3:**
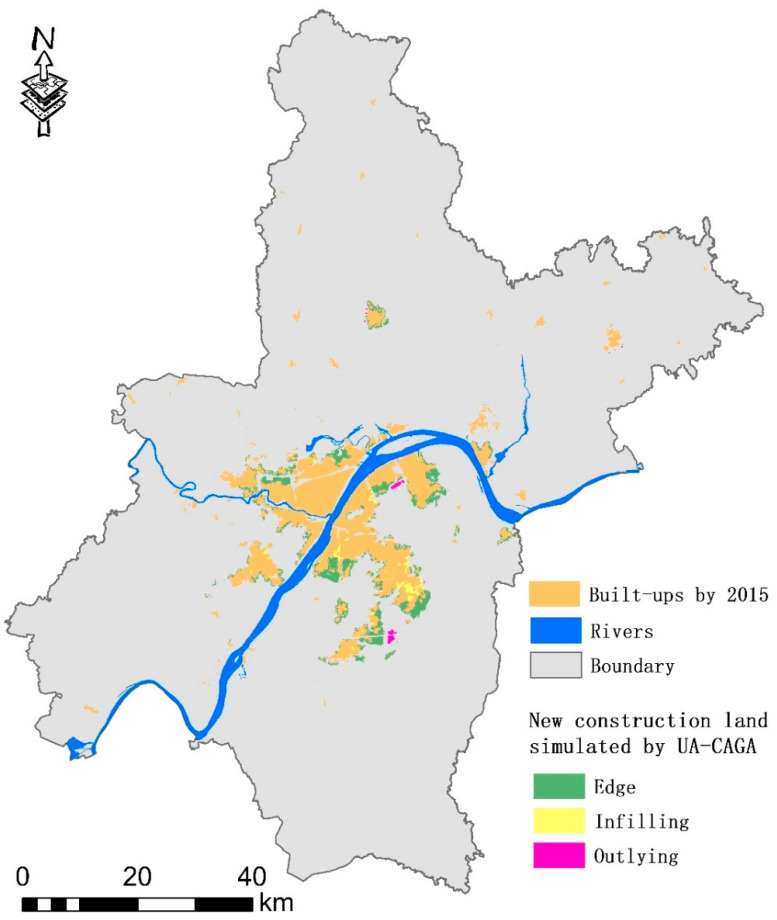
The 2025 spatial distributions of the three simulated expansion types using the UV-CAGA model.

According to statistics for the average urban vitality of different UGP patches, the infilling type had the highest average vitality level (0.215), the edge type had the second highest vitality levels (0.206), and the outlying type had the lowest vitality (0.199). This is consistent with the regression results in Table 3. The spatial distributions of the three types are shown in Figure 3. New construction land patches were located around the largest urban patch in the main urban area. The original construction land was used to create multiple 1 km buffer rings. More than 96.41% of the new construction land area was within 1 km of the main urban area, 3.43% was within 1–2 km, and only 0.16% was outside 2 km. This was consistent with the spatial distribution of city vitality, which declined along the original built-up area. This indicated that UV-CAGA preferentially converted cells with high vitality potential around the main urban area into urban land during optimization. A lack of vitality around the remote urban patches lowered the probability of being “selected” by the model, which differed from the results of other models (see Section 4.1).

## 4. Discussion

### 4.1. Comparison of Optimized and Natural Expansion Forms

**Figure 4:**
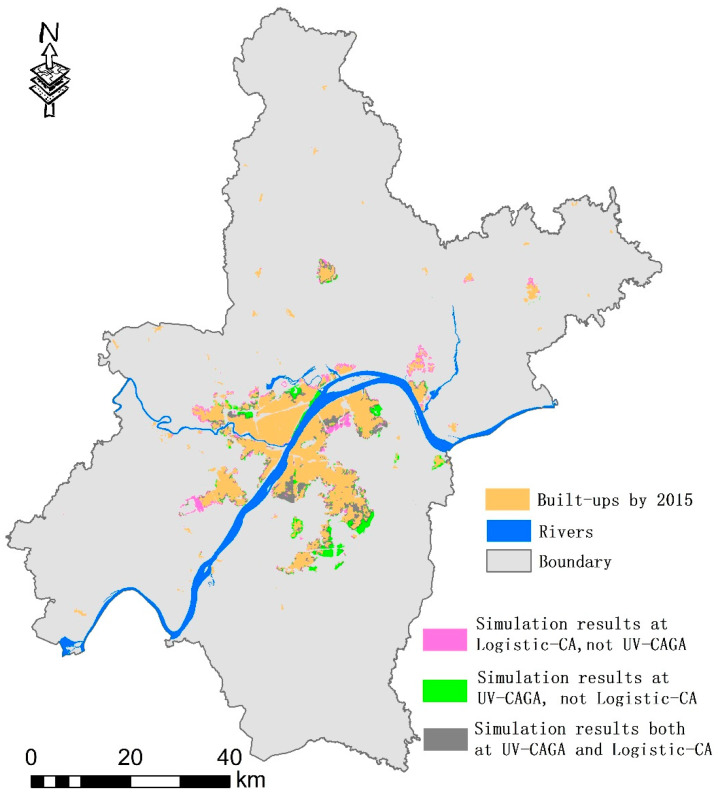
The simulation differences generated by the UV-CAGA and logistic CA models.

Goals provide constraints that can improve urban vitality through the managed evolution of a city’s shape. We used a common urban expansion simulation model, logistic CA, to predict urban evolution from 2015 to 2025 and compared the results with our optimized results. The parameters for the two models were established based on the historical evolution from 2005 to 2015. As a traditional CA model, logistic CA cannot simulate the outlying-expansion process, because of neighborhood rule limitations during cell transformation. The results differed from the urban form generated by the UV-CAGA optimization approach. The average urban vitality values for newly added urban cells in logistic CA were 4.8% lower compared to the optimized result. This showed that urban planning can significantly promote urban vitality by providing guidance for the layout of newly added built-up areas. Furthermore, the results generated by logistic CA were evenly distributed around original construction land patches. This occurred regardless of whether these original construction sites were in the main urban area or in remote areas. In contrast, in the optimization scheme, the newly added construction land was located near the original main urban area, and the distribution in the southeast direction was more concentrated (see Figure 4). To improve vitality, newly added land development should proceed in the southeast.

### 4.2. Generalization of the Model

The urban form determines the spatial configuration of different factors, such as land use, transportation, and population. It also relates to urban environmental security [8], traffic commuting [47,48,49,50], air quality [51,52,53], and other urban spatial phenomena explored by previous scholars. These studies provide solid references for urban form design to reduce traffic congestion, air pollution, resource consumption, and other problems. However, they focus on internal design at the street or block scale and do not consider the optimization of the external macro urban form pattern. We know of no studies that have mined data to conduct correlations between variables, such as urban form and air quality, to simulate the optimization of future urban forms, to explicitly generate an urban form optimization scheme that can promote specific goals. This article proposed an urban morphology optimization model that improves on existing geographic process simulation models and spatial optimization models and involves a coupled “bottom-up” CA model and a “top-down” intelligent GA optimization model. This coupling of research to promote the optimization of the dynamic urban form should help expand traditional CAs that do not simulate the “diffusion” of urban form change. At the same time, the spatial optimization framework in the model’s genetic algorithm provides a framework for other effects related to urban form optimization, such as reducing the concentration of urban air pollutants and promoting urban commuting. Integrating these topics enriches and improves urban planning and related research.

China, India, and other developing countries are currently experiencing rapid urbanization and will continue to improve the rate of urbanization in the future. This provides opportunities to reshape the urban form. Regulating and optimizing the urban spatial form has become a popular research topic [8]. Western developed countries call for and practice the “compact city” concept after experiencing urban sprawl. The UV-CAGA model can explicitly simulate urban growth aggregation, i.e., the compact process, and simulate the urban diffusion process. This provides the optimal allocation of diffusion and polymerization in quantity and space based on goals and constraints. It can be applied both to optimizing the urban form in the context of continuous urban expansion in developing countries and provides a tool for simulating compact urban development and implementing urban renewal in developed countries.

## 5. Conclusions

This paper proposed a model based on improved CA and GA coupling to generate dynamic urban forms. CA was used to model the spatial distribution of different UGP cells. The GA model was based on the goal of promoting vitality, continuously adjusting the direction of the CA spatial model, and generating different optimized UGP cell layout schemes. We first calculated the probability of each cell converting to a different UGP using logistic regression. We then evaluated the factors influencing urban vitality. The main conclusions were as follows:

(1) Modeling cellular transformations to different UGP probabilities showed that being farther from existing construction land significantly promoted outlying evolution. Being closer to the urban and commercial centers led to a higher probability of infilling, with a lower probability of edge development. Population density had a positive effect on edge and infilling, but not outlying evolution. Urban vitality modeling showed that the distance from the urban center and population density had significant positive relationships with urban vitality. However, they had significant negative relationships with distance from the city’s business center, GDP density, distance from bus stops, and road density.

(2) As a diffusion growth mode, outlying evolution accounted for only 4.90% of the newly added urban built-up area; 17.68% occurred through infilling; 77.32% occurred through edge growth. This indicated that the urban form of Wuhan is likely to become very compact in 2025. This suggests that outlying expansion is less likely to promote city vitality compared to the other two types and is less likely to be selected in the optimization process.

(3) Compared with the urban form under natural expansion, the optimized urban form improved vitality by 4.8%. The future urban growth direction should be located southeast of the city.

Similar to all models, the UV-CAGA model does have some limitations. For example, data used for the simulation and optimization of future urban forms extended only back to 2018. The data and values used to evaluate vitality and UGP were static and not dynamic. In contrast, the actual values of these variables, such as population and road density, are constantly changing. Additional research is needed to identify an effective way to simulate changes in these values. Further, future studies could continuously improve the science and accuracy of UV-CAGA model simulation results.

## Figures and Tables

**Table 1:** Urban built-up areas in Wuhan by 1995, 2000, 2005, 2010, 2015, and forecasted area by 2025.

Year	Area (km^2^)
1995	295.85
2000	310.85
2005	371.61
2010	497.02
2015	511.10
2025	643.94

**Table 2:** Three group variable coefficients for edge, outlying, and infilling.

Variables	Edge	Outlying	Infilling
Constant	0.537	−2.495 *	0.38 *
Dis_center	0.104 ***	0.164 **	−0.393 ***
Dis_built	−2.519 **	4.569 ***	−2.857
Dis_rail	0.18	0.077 **	−0.611
Dis_water	−0.115 **	0.019	0.17 ***
Dis_business	0.158 *	0.177 **	−0.813 **
POPD	0.047 **	−0.545 **	0.301 *
RD	0.045 *	−0.042 *	0.064 ***
R2	0.671	0.825	0.842

Notes: ***, ** and * indicate significance levels of 0.01, 0.01 and 0.1, respectively.

**Table 3:** Regression relationship between urban vitality and driving variables.

Variables		Unstandardized Coefficients
${\mathrm{Dis}}_{\mathrm{center}}$		−0.114 **
${\mathrm{Dis}}_{\mathrm{bcenter}}$		−0.198 **
${\mathrm{Dis}}_{\mathrm{station}}$		−0.063
PD		0.039
GDPD		−0.044 *
RD		3.859 **
UGP (Infilling as reference group)	X1(Outlying)	−2463.589 ***
	X2(Edge)	−1054.549 *

Note: * significant level at 0.1; ** significant level at 0.05; *** significant level at 0.01.

## Data Availability

The data presented in this study are available on request from the corresponding author.

## References

[1] He Q., Zhou J., Tan S., Song Y., Zhang L., Mou Y., Wu J. (2019). What is the developmental level of outlying expansion patches? A study of 275 Chinese cities using geographical big data. Cities.

[2] Jacobs J. (1961). The Death and Life of Great America Cities.

[3] Lynch K. (1984). Good City Form.

[4] Huang B., Zhou Y., Li Z., Song Y., Cai J., Tu W. (2019). Evaluating and characterizing urban vibrancy using spatial big data: Shanghai as a case study. Environ. Plan. B Urban Anal. City Sci..

[5] Long Y., Huang C. (2017). Does block size matter? The impact of urban design on economic vitality for Chinese cities. Environ. Plan. B Urban Anal. City Sci..

[6] Rao Y., Dai J., Dai D., He Q. (2021). Effect of urban growth pattern on land surface temperature in China: A multi-scale landscape analysis of 338 cities. Land Use Policy.

[7] He Q., Song Y., Liu Y., Yin C. (2017). Diffusion or coalescence? Urban growth pattern and change in 363 Chinese cities from 1995 to 2015. Sustain. Cities Soc..

[8] Zhou K., Liu Y., Tan R., Song Y. (2014). Urban dynamics, landscape ecological security, and policy implications: A case study from the Wuhan area of central China. Cities.

[9] Rao Y., Yang J., Dai D., Wu K., He Q. (2021). Urban growth pattern and commuting efficiency: Empirical evidence from 100 Chinese cities. J. Clean. Prod..

[10] Sun B., He Z., Zhang T., Wang R. (2015). Urban spatial structure and commute duration: An empirical study of China. Int. J. Sustain. Transp..

[11] Su H., Han G., Li L., Qin H. (2021). How does urban form affect land surface temperature: A case study of 266 Chinese cities from a multi-perspective analysis. Sustain. Cities Soc..

[12] Zhou X., Hong C. (2018). Impact of urbanization-related land use land cover changes and urban morphology changes on the urban heat island phenomenon. Sci. Total. Environ..

[13] He B.-J., Zhao Z.-Q., Shen L.-D., Wang H.-B., Li L.-G. (2018). An approach to examining performances of cool/hot sources in mitigating/enhancing land surface temperature under different temperature backgrounds based on landsat 8 image. Sustain. Cities Soc..

[14] Mou Y., Song Y., Xu Q., He Q., Hu A. (2018). Influence of Urban-Growth Pattern on Air Quality in China: A Study of 338 Cities. Int. J. Environ. Res. Public Health.

[15] Bereitschaft B., Debbage K. (2013). Urban form, air pollution, and CO2 emissions in large US metropolitan areas. Prof. Geogr..

[16] Makido Y., Dhakal S., Yamagata Y. (2012). Relationship between urban form and CO2 emissions: Evidence from fifty Japanese cities. Urban Clim..

[17] Zhao Z.-Q., He B.-J., Li L.-G., Wang H.-B., Darko A. (2017). Profile and concentric zonal analysis of relationships between land use/land cover and land surface temperature: Case study of Shenyang, China. Energy Build..

[18] Yang J., Yang Y., Sun D., Jin C., Xiao X. (2021). Influence of urban morphological characteristics on thermal environment. Sustain. Cities Soc..

[19] Luo X., Yang J., Sun W., He B. (2021). Suitability of human settlements in mountainous areas from the perspective of ventilation: A case study of the main urban area of Chongqing. J. Clean. Prod..

[20] Yang J., Ren J., Sun D., Xiao X., Xia J., Jin C., Li X. (2021). Understanding land surface temperature impact factors based on local climate zones. Sustain. Cities Soc..

[21] Honey-Rosés J., Anguelovski I., Chireh V.K., Daher C., Bosch C.K.V.D., Litt J.S., Mawani V., McCall M.K., Orellana A., Oscilowicz E. (2020). The impact of COVID-19 on public space: An early review of the emerging questions—Design, perceptions and inequities. Cities Health.

[22] Fabris L.M.F., Camerin F., Semprebon G., Balzarotti R.M. (2020). New Healthy Settlements Responding to Pandemic Outbreaks: Approaches from (and for) the Global City 2020. Plan J..

[23] Slater S.J., Christiana R.W., Gustat J. (2020). Recommendations for Keeping Parks and Green Space Accessible for Mental and Physical Health During COVID-19 and Other Pandemics. Prev. Chronic Dis..

[24] Florida R., Rodríguez-Pose A., Storper M. (2021). Cities in a post-COVID world. Urban Stud..

[25] He Q., He W., Song Y., Wu J., Yin C., Mou Y. (2018). The impact of urban growth patterns on urban vitality in newly built-up areas based on an association rules analysis using geographical ‘big data’. Land Use Policy.

[26] Wu J., Ta N., Song Y., Lin J., Chai Y. (2018). Urban form breeds neighborhood vibrancy: A case study using a GPS-based activity survey in suburban Beijing. Cities.

[27] Delafons J. (1991). The New Urbanism: Toward an Architecture of Community.

[28] Fulton W. (1996). The New Urbanism: Hope or Hype for American Communities?.

[29] Jin X., Long Y., Sun W., Lu Y., Yang X., Tang J. (2017). Evaluating cities’ vitality and identifying ghost cities in China with emerging geographical data. Cities.

[30] Tang L., Lin Y., Li S., Li S., Li J., Ren F., Wu C. (2018). Exploring the Influence of Urban Form on Urban Vibrancy in Shenzhen Based on Mobile Phone Data. Sustainability.

[31] Song Y. (2005). Smart Growth and Urban Development Pattern: A Comparative Study. Int. Reg. Sci. Rev..

[32] Resnik D. (2010). Urban sprawl, smart growth, and deliberative democracy. Am. J. Public Health.

[33] Wu J., Wu Y., Yu W., Lin J., He Q. (2019). Residential landscapes in suburban China from the perspective of growth coalitions: Evidence from Beijing. J. Clean. Prod..

[34] Thomas L., Cousins W. (1996). The compact city: A successful, desirable and achievable urban form. Compact. City A Sustain. Urban Form.

[35] Ewing R., Cervero R. (2010). Travel and the built environment: A meta-analysis. J. Am. Plan. Assoc..

[36] Punter J., Yu L., Ye J. (2005). Sustainable Principles of Urban Development Pattern. Urban Plan. Int..

[37] Liu X., Ma L., Li X., Ai B., Li S., He Z. (2013). Simulating urban growth by integrating landscape expansion index (LEI) and cellular automata. Int. J. Geogr. Inf. Sci..

[38] Liu Y., He Q., Tan R., Liu Y., Yin C. (2016). Modeling different urban growth patterns based on the evolution of urban form: A case study from Huangpi, Central China. Appl. Geogr..

[39] Castella J.-C., Kam S.P., Quang D.D., Verburg P., Hoanh C.T. (2007). Combining top-down and bottom-up modelling approaches of land use/cover change to support public policies: Application to sustainable management of natural resources in northern Vietnam. Land Use Policy.

[40] Yuan M., Liu Y. (2014). Land use optimization allocation based on multi-agent genetic algorithm. Trans. Chin. Soc. Agric. Eng..

[41] Li X., Chen Y., Liu X., Xu X., Chen G. (2017). Experiences and issues of using cellular automata for assisting urban and regional planning in China. Int. J. Geogr. Inf. Sci..

[42] Tan R., Liu Y., Zhou K., Jiao L., Tang W. (2015). A game-theory based agent-cellular model for use in urban growth simulation: A case study of the rapidly urbanizing Wuhan area of central China. Comput. Environ. Urban Syst..

[43] Dougal C., Parsons C.A., Titman S. (2015). Urban Vibrancy and Corporate Growth. J. Finance.

[44] Chi G., Liu Y., Wu Z., Wu H. (2015). Ghost cities analysis based on positioning data in China. arXiv.

[45] Liu X., Li X., Chen Y., Tan Z., Li S., Ai B. (2010). A new landscape index for quantifying urban expansion using multi-temporal remotely sensed data. Landsc. Ecol..

[46] de Koe D.M., HvA P.D.B. (2015). Urban vitality through a mix of land-uses and functions: An addition to citymaker. Ph.D. Dissertation.

[47] Engelfriet L., Koomen E. (2017). The impact of urban form on commuting in large Chinese cities. Transportation.

[48] Marcinczak S., Bartosiewicz B. (2018). Commuting patterns and urban form: Evidence from Poland. J. Transp. Geogr..

[49] Li X., Mou Y., Wang H., Yin C., He Q. (2018). How Does Polycentric Urban Form Affect Urban Commuting? Quantitative Measurement Using Geographical Big Data of 100 Cities in China. Sustainability.

[50] Hu L., Sun T., Wang L. (2018). Evolving urban spatial structure and commuting patterns: A case study of Beijing, China. Transp. Res. Part D Transp. Environ..

[51] Yuan M., Song Y., Huang Y., Hong S., Huang L. (2017). Exploring the Association between Urban Form and Air Quality in China. J. Plan. Educ. Res..

[52] Yuan M., Huang Y., Shen H., Li T. (2018). Effects of urban form on haze pollution in China: Spatial regression analysis based on PM2.5 remote sensing data. Appl. Geogr..

[53] Lee C. (2019). Impacts of urban form on air quality: Emissions on the road and concentrations in the US metropolitan areas. J. Environ. Manag..

